# The mismeasure of ape social cognition

**DOI:** 10.1007/s10071-017-1119-1

**Published:** 2017-08-04

**Authors:** David A. Leavens, Kim A. Bard, William D. Hopkins

**Affiliations:** 1 School of Psychology, University of Sussex, Falmer, Brighton, BN1 9QH UK; 2 University of Portsmouth, Portsmouth, UK; 3 Georgia State University, Atlanta, GA USA

**Keywords:** Social cognition, Mental causality, Comparative methods, Species comparisons

## Abstract

In his classic analysis, Gould (The mismeasure of man, WW Norton, New York, 1981) demolished the idea that intelligence was an inherent, genetic trait of different human groups by emphasizing, among other things, (a) its sensitivity to environmental input, (b) the incommensurate pre-test preparation of different human groups, and (c) the inadequacy of the testing contexts, in many cases. According to Gould, the root cause of these oversights was confirmation bias by psychometricians, an unwarranted commitment to the idea that intelligence was a fixed, immutable quality of people. By virtue of a similar, systemic interpretive bias, in the last two decades, numerous contemporary researchers in comparative psychology have claimed human superiority over apes in social intelligence, based on two-group comparisons between postindustrial, Western Europeans and captive apes, where the apes have been isolated from European styles of social interaction, and tested with radically different procedures. Moreover, direct comparisons of humans with apes suffer from pervasive lapses in argumentation: Research designs in wide contemporary use are inherently mute about the underlying psychological causes of overt behavior. Here we analyze these problems and offer a more fruitful approach to the comparative study of social intelligence, which focuses on specific individual learning histories in specific ecological circumstances.

## Hereditarianism and human intelligence

A century ago, developers of intelligence tests were convinced that they had devised measures of native intellectual ability in our species (Gould 1981). By mid-century, it was apparent that performance on these assessments was highly influenced by non-hereditary factors, and they were re-interpreted as effective predictors of performance, rather than as instruments that revealed something essential about mental aptitude (for review see Neisser et al. 1996). We now know that intellectual performance in our species is a function of gene–environment interactions: impoverished environments have systematically deleterious effects on mental development (Nelson et al. 2007). Yet, today, researchers routinely report, in the most prestigious journals, claims that human children, even as young as 12 months of age, are inherently superior in social intelligence—the skilled negotiation of social interactions—to our nearest living relatives, the great apes. Of particular relevance to this special issue is the fact that a large proportion of this literature derives from the study of apes’ use and comprehension of gestures. Here we resurrect Gould’s (1981) classic analysis of the misuse of so-called intelligence tests in humans and apply these principles to the contemporary literature on alleged ape–human differences in social intelligence. We also deconstruct the logical pathways from research design to conclusions in a number of representative studies, demonstrating the widespread infiltration of fallacious reasoning in this field of endeavor. We conclude with specific recommendations for more legitimate research design and reasoning in comparative cognition.

In examining Yerkes’s (1921) monograph on intelligence testing in military recruits, Gould (1981) noted that, among other variables, health and schooling were correlated with IQ. For example, military recruits infested with hookworm performed reliably worse than non-infected recruits. Similarly, military recruits with less formal schooling experience performed worse than recruits with more schooling. Variations in IQ were, thus, variable in relation to environmental factors. Yerkes interpreted these variations as gene-environment correlations—in effect arguing that more intelligent people were more effective in avoiding hookworm and more persistent in pursuing schooling. A colleague of Yerkes, Carl Brigham (1923, cited in Gould 1981), when faced with a sharp north/south divide in mental test scores in Black recruits even argued that more intelligent Black people migrated to the northern USA to benefit from the northern states’ increased school expenditure. Thus, even obvious effects of environmental factors on mental performance were ignored in favor of nativist explanations for systematic group differences.

Gould (1981) also excoriated Brigham for ignoring important differences in recruits’ preparations for taking these early IQ tests. Brigham found a consistent, large advantage on psychometric tests of intelligence for longer-term residents of the USA than for recent arrivals. Rather than accepting the obvious explanation that people longer resident in the USA had more familiarity with American customs and the English language, Brigham proffered a tortuous argument to the effect that the composition of immigrants from Europe had shifted from more intelligent and creative people of northern European descent toward people of Slavic or southern European origins in the years immediately prior to testing. This kind of obvious bias and special pleading toward nativist explanations for systematic group differences in test preparedness seems antiquated to contemporary scientists, but as we shall see, it is entirely characteristic of cross-species comparisons between humans and apes. (Brigham 1930, later recanted these nativist conclusions, noting that they were “without foundation,” p 165.)

Finally, Gould (1981) observed the profound lack of standardization in selection criteria and administrative protocols across different venues of the early military tests. In short, there was no standardization of sampling criteria across different human groups, nor was there standardization of testing protocols across these groups—these pervasive confounds with “race” were generally ignored by what Gould termed the “hereditarians”; a cadre of intellectuals and scientists who were committed, a priori, to the idea that there were systematic differences between different “kinds” of people—for example, it was self-evident to these researchers that northern Europeans were more intellectually able than southern or eastern Europeans. Hence, blinded by their commitment to this assumption of northern European superiority, the researchers turned a blind eye to the lack of standardization in sampling regimens and testing conditions that pervaded and confounded group identity with sampling and measurement procedures. We argue, here, that a parallel, systemic blindness exists in the contemporary practice of comparative cognition: Virtually no attempt is made to exert control over sampling criteria from different species, and only rarely are even similar protocols used to test representatives of different species, especially when one of those species is human (Bard and Leavens 2009, 2014; Hopkins et al. 2013; Leavens 2014; Leavens and Bard 2011; Leavens et al. 2008; Lyn 2010; Lyn et al. 2010; Racine et al. 2008). Because one of the most oft-stated ambitions of research in comparative cognition is to chart the taxonomic distribution of cognitive character states (e.g., Povinelli and Eddy 1996, pp 1–16; and the special section on comparative cognition in the journal, *Psychological Science* 1993, vol. 4, iss. 3, among others), it is of significant theoretical import to evaluate the state of the art in this field. One of the goals of this comparative research program is to reveal the existence of alleged specialized learning mechanisms which can, in turn, inform theoretical considerations of the selective histories in different taxonomic lineages. As will become clear, below, we believe that there are significant and widespread methodological and logical deficiencies with much contemporary work in this area, and this has substantially skewed theoretical consideration of these selective pressures to relatively recent times—particularly ecological changes of our ancestors during the Pliocene and Pleistocene epochs, in which no existing congener to our own species can claim their origin (the last common ancestor of apes and humans existed in the Miocene). Thus, there is a suite of allegedly unique human cognitive specializations for understanding other minds that have, according to an increasingly dominant view in comparative psychology and in ethology, no parallel in the other hominoids. This dominant view in contemporary theory, that there is no substantive psychological continuity between humans and their closest living relatives, has focused theoretical attention on the presumed selective effects of the ecological circumstances of Plio-Pleistocene hominids. Moreover, these cross-species psychological assessments are often presented as unbiased assays of real, systematic species differences in psychological function that neatly discriminate between the taxa under scrutiny. Here we argue that the design of many of these assays of psychological function cannot support any unqualified assertion to the effect either (a) that such data clearly identify psychological mechanisms or (b) that these comparative studies between humans and other animals have identified psychological discontinuities between the species, grounded in evolutionarily adaptive events or processes.

## Confounded research designs

Some readers might believe that we are over-stating the case; after all, is not there nearly a scientific consensus that apes have difficulty following human cues, such as gaze and pointing, to locations of hidden food? Table 1 lists a number of representative studies that directly compared apes with human children, and all reported an advantage to humans in the cognitive capabilities allegedly under test. This tabulation reveals that testing environments, pre-experimental task-relevant preparation, sampling protocols, testing protocols, and age at testing were all systematically confounded with species classification, exactly paralleling the pervasive deficiencies of the intelligence testing protocols of a century ago, as identified by Gould (1981). In all of the studies in Table 1, the apes were tested in cages, whereas the humans were not tested in cages—there were, thus, systematic differences in testing environments, and none of these studies made any apparent attempt to match testing environments across the groups. To accept the reported findings at face value, a reader must assume that engaging participants through cage bars or cage mesh has no effect on performance, an assumption that is unwarranted (see Kirchhofer et al. 2012, for evidence of the suppressive effect of physical barriers on performance in dogs, *Canis familiaris*).

**Table 1:** Representative claims of evolutionarily based human uniqueness in social cognition based on direct ape–human comparisons are confounded with systematic group differences in testing environment, task preparation, sampling protocols, testing procedures, and/or age of subjects at testing

Source	Putative mental state (*p*)^a^	Confounds (Y = present, N = absent)				
		Envir.^b^	Task Prep.^c^	Sampl.^d^	Test. Proc.^e^	Age
Povinelli and Eddy (1996)^f^	Seeing leads to knowing	Y	Y	Y	Y	N
Povinelli et al. (1997)^g^	Appreciation of internal mental focus	Y	Y	Y	Y	Y
Tomasello et al. (1997)^h^	Understanding communicative intentions	Y	Y	Y	Y	Y
Call and Tomasello (1999)^i^	Understanding false belief	Y	Y	Y	Y	Y
Povinelli et al. (1999)^j^	Understanding attention as a mental state	Y	Y	Y	Y	Y
Warneken et al. (2006)^k^	Shared intentionality	Y	Y	Y	Y	Y
Herrmann et al. 2007^1^	Understanding communicative intentions	Y	Y	Y	Y	Y
Liszkowski et al. 2009^m^	Common conceptual ground	Y	Y	Y	Y	Y
van der Goot et al. (2014)^n^	Common conceptual ground	Y	Y	Y	Y	Y

Refutations: Most of these studies, except Povinelli et al. (1999), asserted a theoretical rationale comprising a major premise of the form: if *p* then *q*, and reported an absence of a behavior (~*q*) in apes, and therefore, refutations are empirical demonstrations that these index behaviors have been displayed by apes (*q*); Povinelli et al. (1999) asserted that if organisms understood visual attention at a high level (*p*), then they expected the absence of a discrimination of gaze direction in their probe condition C (~*q*)—in their study humans failed to discriminate gaze direction (~*q*), but chimpanzees did discriminate gaze direction (*q*), therefore the refutation by Thomas et al. (2008) involved the demonstration by *reductio ad absurdum* that human adults discriminated gaze direction (*q*) like the chimpanzees in Povinelli et al. (1999), and therefore, according to the argument of Povinelli and colleagues, human adults displayed a low-level, non-mentalistic understanding of visual attention

^a^*p* = antecedent in the conditional: if *p* then *q*

^b^*Envir*. Testing environment

^c^*Task Prep*. Task preparation (i.e., pre-experimental, task-relevant experience)

^d^*Sampl*. sampling procedure

^e^*Test. Proc*. Testing procedure

^f^Refuted by Bulloch et al. (2008), Hostetter et al. (2007)

^g^Refuted by Lyn et al. (2010), Mulcahy and Call (2009)

^h^Partially refuted (pointing comprehension) by Lyn et al. (2010), Mulcahy and Call (2009)

^i^refuted by Krupenye et al. (2016)

^j^Refuted by Thomas et al. (2008)

^k^Refuted by Bard et al. (2014a), Warneken et al. (2007)

^l^Refuted by Russell et al. (2011)

^m^Refuted by Bohn et al. (2015, 2016), Lyn et al. (2014)

^n^Refuted by Leavens et al. (2015)

In all of the studies listed in Table 1, institutionalized apes were compared with non-institutionalized human children. The vast majority of the apes involved were isolated from early intensive exposure to human nonverbal conventions of give and take and of daily exposure to nonverbal reference to entities, whereas none of the human children were so isolated. Hence, the human children had had extensive task-relevant preparation when challenged to use human nonverbal cues, such as ostensive gaze or pointing gestures, for example, to find hidden objects. Yet the researchers cited in Table 1 universally concluded that the humans’ superior performances were attributable to their evolutionary, and not their developmental histories. Even when the apes outperformed humans, as in Povinelli et al. (1999), the scientists interpreted their chimpanzees’ (*Pan troglodytes*) superior performance as evidence for the animals’ inferior understanding of visual attention. In some of these studies, we find a few individual apes who had been enculturated from an early age. For example, in Tomasello et al. (1997), two of the apes, Chantek, an orangutan (*Pongo pygmaeus*), and Erika, a chimpanzee, had been raised in human cultural environments, whereas the remaining apes in that study were institutionalized from birth. When computing the average performance of the apes with the human children (two-and-a-half to 3 years old), the authors found a statistically significant difference between humans and apes in using a pointing gesture to find hidden objects, favoring the humans. But when Leavens (2014) compared the children with the only two apes who had had commensurate task-relevant preparation, he found that the apes performed comparably. Thus, as Leavens (2014) noted, there is a systematic and methodologically problematic tendency to artificially suppress the results of non-humans by averaging performance data between (a) apes that have had significant task-relevant preparation (enculturated apes) and (b) apes that have been denied this preparation (institutionalized or sanctuary-housed apes). Thus, in order to accept these reports that apes have difficulty understanding deictic gestures or producing deictic gestures, a reader must assume that experience with the daily use of deictic gestures is not relevant to performance in understanding or producing deictic gestures—an unwarranted assumption (Leavens 2006; Leavens et al. 2008; Lyn 2010; Lyn et al. 2010).

A related and systematic confound with species classification is sampling protocol. It is rudimentary that failure to match sampling protocols introduces a confound into any group comparison. Thus, for an example, Kirchhofer et al. (2012) compared pet dogs with chimpanzees in their understanding of experimenters’ pointing gestures—the specific task was that the subjects were to fetch the objects to which experimenters pointed. As noted by Hopkins et al. (2013), the dogs were recruited through advertisements, introducing a self-selection procedure for the dog owners, whereas the apes were opportunistically sampled from a zoo and from a sanctuary. Although Kirchhofer et al. interpreted the superior performance by the dogs as evidence for the effects of artificial selection (breeding), in fact, it is ambiguous whether the selective histories or the different sampling protocols account for the group differences observed. For another example, Liszkowski et al. (2009) selected only human infants who had demonstrated prior use of pointing, but did not apply this same selection criterion to their ape subjects. Similarly, with reference to the studies listed in Table 1, where apes were compared to humans, the apes were always opportunistically sampled from captive populations, whereas the children were recruited, primarily, through advertisements. It is categorically ambiguous, therefore, whether the group differences reported in the papers listed in Table 1 are attributable to systematic differences in evolutionary histories or to differences in sampling protocols.

With respect to test procedures, none of the studies listed in Table 1 administered the same procedures to the apes and to the humans. For example, Povinelli and Eddy (1996) were unable to teach human two- to seven-year-old children to point to experimenters (p 109, fn 6),^1^ so instead of requiring the same gestural response from the children and the apes that were compared in that study, the authors required the children to indicate their choice of experimenter by placing their hands on a handprint provided for them. Here, the experimenters were unable to elicit pointing gestures from human children, but claimed cognitive superiority for these same children, compared with apes who pointed to an experimenter through a hole in a plexiglas barrier. In that study, it is ambiguous whether the children outperformed the apes because of their alleged cognitive superiority (as Povinelli and Eddy claimed) or because the experimenters administered an easier task to the human children. For another example, van der Goot et al. (2014) measured whether apes and human children locomoted to the closest possible proximity to unreachable toys (human children) or food (mostly adult apes), but the humans were tested at distances of less than 2 m between themselves and the unreachable toys, whereas the apes were (inexplicably) presented with unreachable food at a distance of 6 m. They found that roughly half of the children stayed in situ and pointed to the toys without locomoting to proximity with the toys, whereas none of the 10 apes gestured to food without first traversing the 6 m to the closest proximity with the food before gesturing. van der Goot and her colleagues interpreted this group difference as evidence for a uniquely human capacity to discern a state of psychological common ground, but because two different procedures were administered to the two groups (apes and human children), it is unclear whether the apes were more likely than the children to move to proximity with the unreachable entities because of their evolutionary histories or because of some or all of the many systematic procedural differences. In an observational study, Leavens et al. (2015) found that when they presented unreachable food to 166 chimpanzees at distances approximating those used with human infants in van der Goot et al., then like the children in van der Goot et al., approximately half of the apes communicated from a distance, and half moved to proximity with the food before signaling about it (note that approximately matching just one procedural feature, distance, led to statistically indistinguishable response profiles between humans and apes, despite many procedural differences between Leavens et al. 2015 and van der Goot et al. 2014). Thus, all direct ape–human comparisons that have reported human superiority in cognitive function have universally failed to match the groups on testing environment, test preparation, sampling protocols, and test procedures, including those that tested subjects’ comprehension and production of communicative gestures (Table 1), although we provide only a few examples, here.

Moreover, as repeatedly noted by Bard and her colleagues (e.g., Bard and Leavens 2014; Bard et al. 2014a), none of these studies matched the apes with the humans on age at testing (Table 2); indeed, in only one of these studies, that by Povinelli and Eddy (1996), was there even any overlap in age between the apes and the humans. For example, Liszkowski et al. (2009) compared 12-month-old human children with apes that were, on average, 19 years old, reporting that humans, but not apes, communicated about absent entities. van der Goot et al. (2014) compared 12-month-old human children with apes that were, on average, nearly 18 years old, concluding that humans, but not apes, communicate with gestures from a distance. Again, it is ambiguous whether the group differences reported by these authors cited in Table 1 are attributable to differences in evolutionary histories, as the authors claimed, or to the systematic differences in life history stage at which these subjects were tested—not one of these studies validated their protocols on humans that were age-matched to the apes, again, with the possible exception of Povinelli and Eddy (1996).

**Table 2:** Age differences at time of testing are confounded with species classifications in direct ape–human comparisons: representative studies

	Age ranges in years (*n*)		
	Humans	Apes	Overlap in age?
Povinelli and Eddy (1996)	2–7 (47)	4–6 (6)	Yes
Povinelli et al. (1997)			
Experiment 1	3 (24)	6–7 (7)	No
Experiment 2	3 (12)	6–7 (7)	No
Tomasello et al. (1997)	2.5–3 (48)	7–23 (8)	No
Call and Tomasello (1999)	4–5 (28)	7–37 (9)	No
Povinelli et al. (1999)	3 (24)	6 (7)	No
Warneken et al. (2006)	1.5–2 (32)	3–4 (3)	No
Herrmann et al. (2007)	2 (105)	3–21 (138)	No
Liszkowski et al. (2009)	1 (32)	6–31 (16)	No
van der Goot et al. (2014) (Exper. 1)	1 (20)	9–35 (16)	No

Mean ages are not computable for all of these studies, and hence we report minimum and maximum ages at times of testing for each group

*Exper*. Experiment

These studies (Table 1) failed to control for systematic group differences in environment, task preparation, sampling protocols, testing procedures, and age, yet these researchers not only concluded, often implicitly, that these confounds were irrelevant, through asserting that species classification (i.e., evolutionary history) was the only relevant factor, they also managed to convince a number of reviewers and editors that these confounds were not relevant to their findings of group differences. The journals in which the papers listed in Table 1 were not, by and large, obscure journals: they included *Science*, *Psychological Science*, and *Child Development*—prestigious journals with large international readerships. Thus, manifestly, reviewers and editors at some of the most influential scientific journals believed that these researchers had identified an influence of evolutionary history on the cognitive underpinnings of, among other things, apes’ and humans’ understanding and production of communicative gestures. Yet, when we cursorily examine some of the uncontrolled variables in these studies, we find that not one of these papers has isolated evolutionary history as the singular factor in the group performance differences reported in these papers. This pervasive collapse in experimental and interpretive rigor is not unprecedented, as Gould (1981) so elegantly noted in relation to the virtually unquestioned assumption, 100 years ago, that northern Europeans had the highest average intellectual capacity in our species. This tacit understanding seemed to have the effect of “blinding” researchers to the multitudinous confounds that existed alongside their racial group classifications. Our reading of the contemporary literature on comparative social cognition leads us to assert that there are similarly numerous and universal confounds of method with species classifications. It is our contention, here, that there is no methodologically sound report of an essential difference between apes and humans in their abilities to use or comprehend simple gestural cues, due to the systematic confounds listed in Table 1. This is not to claim that there could not be a such a demonstration in the future, but it seems clear from the many uncritical citations of alleged ape–human differences in the ability to use and comprehend simple deictic gestures, like overt gaze and pointing, that many contemporary researchers have abandoned any critical evaluation of these empirically unfounded claims. It seems possible, in view of the chasm that exists between evidence and belief that we document here, that there may be a deep, yet unwarranted commitment to the ideas (a) that comprehension and production of pointing, understanding of visual attention, understanding common ground, or discrimination of false belief require sophisticated reasoning abilities and (b) that humans uniquely possess these hypothetical reasoning abilities.

## The scientific sterility of two-group, two-species comparisons

In addition to the systematic methodological weaknesses that underlie reports of human superiority in the use and understanding of simple directional gestures, these claims rely on the core assumption that intentional and epistemic states cause overt behavior. This model of mental cause with behavioral effect is scientifically unfalsifiable whenever the putative cause is not empirically measurable.

It is a near-universal premise in the contemporary cognitive sciences that mental states cause behavior.^2^ While there are many critiques of this premise, including theoretical positions grounded in distributed or embodied cognitive perspectives (e.g., Barrett 2015; Chemero 2011; Johnson 2001; Sehon 2000; Varela et al. 1991) and also some recent extensions of behaviorism, in which contingencies are conceived of as having very extended temporal manifestations (including Baum’s *molar* behaviorism and Rachlin’s *teleological* behaviorism; see, e.g., Baum 2002; Rachlin 1992),^3^ here we will establish the unfalsifiability of this premise to illustrate the logic used in the many claims of human uniqueness. As noted by Malle and Hodges (2005), there are classes of mental state that can be effects of behavior (e.g., perceptions) and there are classes of mental state that are causes (e.g., epistemic states). It is this latter class of hypothetical mental states that concerns us, here—including intentions, beliefs, and desires.

A representative range of putative causal mental states are presented in Table 1. There are, in broad terms, two versions of the mental causality model: a strong version in which particular behavioral patterns (*q*) index particular causal mental states (*p*)—that is, the intentional or epistemic state is both necessary and sufficient to cause the behavior pattern—and a weaker version in which a particular behavioral pattern merely implicates a particular mental state—in other words, the putative mental state is a sufficient, but not a necessary condition for display of the behavior pattern of interest.

## Mental states as necessary causes (biconditional)

An example of the claim that some gestures index particular causal mental states is that by Tomasello et al. (e.g., 2007); in their account, declarative-expressive and declarative-informative gestures index a psychological appreciation of others’ minds. Declarative-expressive gestures, according to this account, include such acts as pointing to an entity with the motive that an interlocutor share attention to that entity: “the communicator wants the recipient to feel some attitude or emotion that he is already feeling” (p 707). Declarative-informative gestures, on the other hand, allegedly index a motivation for sharing states of knowledge—for example, a child might point to inform an interlocutor of the location of hidden entities, or as Tomasello et al. (2007) put it: “the communicator wants the recipient to know something that he thinks she will find useful or interesting” (p 707). According to proponents of this view, first, the mere fact of declarative-expressive and declarative-informative communication constitutes evidence for these communicative motivations that allegedly couple the emotions or the epistemic states of two interlocutors, and, second, these motivations are absent in humans’ nearest living relatives, the great apes:

apes do not produce, either for humans or for other apes, points that serve functions other than the imperative/requestive function. That is, they do not point declaratively to simply share interest and attention in something with another individual, and they do not point informatively to inform others of things they want or need to know (Tomasello et al. 2007, p 717).

Thus, in this strong version of the mental causality model, the alleged absence of declarative-expressive and declarative-informative gestures in great apes entails that apes lack these putative cognitive states, and the presence of declarative-expressive and declarative-informative gestures in our species entails that humans possess these mental states.

According to the logic of necessary and sufficient causes (if *p* then *q* AND if *q* then *p*, or *p* ⇔ *q*), the biconditional relationship between *p* and *q* is true only if *p* and *q* are always both true (present) or both false (absent; Table 3). Thus, if these putative communicative motivations to share emotional and epistemic states (*p*) occur in the absence of declarative-expressive or declarative-informative communication (*q*), then the claim that these hypothetical mental states are necessary and sufficient for declarative communication is falsified. Also, if declarative-expressive or declarative-informative communication (*q*) occurs in the absence of the motivation to share emotional or epistemic states (*p*), then the postulate of the biconditional relationship between the alleged causal mental states and the diagnostic communicative behaviors is falsified.

**Table 3:** Biconditional mental causality models, and their implications for comparative cognition

Assume *p* exists			The reality: *p* is indeterminable		
*p*	*q*	*p* ⇔ *q*	*p*	*q*	*p* ⇔ *q*
Biconditional (*p* is a necessary cause for *q*; *q* if and only if *p*)					
T	T	T	T?	T	?
T	F	F*	T?	F	?
F	T	F*	F?	T	?
F	F	T	F?	F	?

Under the assumption, at left, that *p* is determinable (i.e., that the presence and absence of *p* is determinable, hence a truth value can be legitimately applied to both *p* and *q*), the asterisks denote the states of the world that would disconfirm the premise *p* ⇔ *q*. The premise *p* ⇔ *q* would be falsified whenever *p* and *q* have incommensurate truth values (i.e., whenever one is true, or present, and the other is false, or absent). Here we argue that, in reality, because *p* is imaginary and cannot be objectively measured, as shown at right, therefore there is no possibility of disconfirming the premise *p* ⇔ *q*. Thus, all mental causality models that posit a certain mental state (*p*) to be a necessary cause for a particular behavior (*q*) are unfalsifiable

That this strong version of the mental causality model of gestural communication is empirically unfalsifiable is obvious when one reflects that it is, in practice, impossible to assign a truth value to the presence or absence of any hypothetical causal mental state, *p* (e.g., Bergmann 1962; Leavens et al. 2004a; Vanderwolf 1998). Thus, although declarative-expressive and declarative-informative communicative acts can be either present or absent, empirically, because we cannot objectively measure the presence and absence of the putative causal mental state (*p*), therefore, the claim that declarative-expressives and declarative-informatives (*q*) uniquely implicate these alleged causal mental states is not a scientifically falsifiable claim. This might not be immediately obvious to some readers, but if, instead of causal mental states, we were to argue that demonic possession entailed declarative-expressive and declarative-informative communicative acts, then it should be straightforward to see that the association between demonic possession and communicative acts cannot be empirically determined—there is no such thing as a demonic possession measuring device, notwithstanding the widely held belief that supernatural agents influence human behavior.^4^

Moreover, there are many published examples of declarative-expressive and declarative-informative communicative acts performed by great apes (for reviews, e.g., Leavens and Bard 2011; Leavens et al. 2008; Leavens and Racine 2009):

both declarative and informative pointing have been reported in apes, usually, but not always, language-trained or home-raised apes. Examples of informative pointing include Peter, who when asked, “Where’s Dada?” pointed to Mr. McArdle (Witmer 1909); Gua, who pointed to her nose when asked, “Where is your nose?” and who pointed to pictures of objects when given their names (Kellogg and Kellogg 1933); Washoe, who often pointed in response to similar queries, but who was often further required to sign the object’s name (see discussion in Savage-Rumbaugh et al. 1985); Matata, who sometimes pointed in the direction of faraway noises while walking in the woods (Savage-Rumbaugh et al. 1998); and Kanzi, Panbanisha, and Nyota, who pointed declaratively and informatively (Pedersen et al. 2009). In short, virtually every language-trained or home-raised ape apparently points declaratively or informatively (for example, in response to questions of the form, “Where is X?”) (Leavens and Bard 2011:18).

Declarative-informative pointing by a single, free-ranging bonobo (*Pan paniscus*) was reported by Veà and Sabater-Pi (1998), and an apparent declarative-expressive deictic gesture by a wild chimpanzee was reported by Hobaiter et al. (2014). The showing of an object by a young chimpanzee to their social partner was reported by Russell et al. (1997); this was a quintessential, declarative-expressive signal, as defined for human children by Bates et al. (1975). Thus, declarative-expressive and declarative-informative communicative acts have been reported in great apes, and therefore, according to the biconditional argument of Tomasello et al. (2007), that these behaviors index certain causal mental states, we must attribute to these animals the communicative motivations that they claim are signified by this kind of behavior, the motivations to share feelings and to share epistemic states. In other words, if the major premise *p* ⇔ *q* is assumed to be true, as asserted by Tomasello et al. (e.g., 2007), then the demonstration of *q* in non-humans entails the presence and causal influence of the hypothetical mental state *p*. No other interpretation is possible with a strong mental causality model that posits unique behavioral indices of causal mental states (see Table 3). However, rather than defend their own postulate, and acknowledge that according to their own psychological process model, great apes share with humans the motivations to share feelings and epistemic states, Tomasello and his colleagues have taken the position that such reports constitute measurement error. That is, they argue that the existing reports of declarative signals displayed by non-human primates and other animals constitute misclassifications of behavior, and they include as an example of such misclassification their own previous report of declarative communication by two chimpanzees (Carpenter et al. 1995; see discussion in Carpenter and Call 2013).

It becomes clear that a strong version of the mental causality model of gestural communication is untenable when the nature of the measurement error (misclassification) is specified. Thus, Carpenter and Call (2013) argued that “when apes gesture for others, there is no unequivocal evidence that they do so with the sole (and spontaneous) goal of sharing attention and interest with others about something” (p 57). This position contains two subtle rhetorical devices: a misdirection and a begging of the question. First, the reader is misdirected toward a focus on the psychological ambiguity of ape gestures and away from the commensurate ambiguity of young humans’ gestures; because there is no “unequivocal evidence” that any nonverbal organism of any species ever displays the “sole (and spontaneous) goal of sharing attention and interest,” therefore the strong mental causality model reduces to a simple interpretive bias to the effect that if the signaler has a lot of fur, then the communicative act is not performed with the hypothetical mental cause (*p*). Second, Carpenter and Call conclude that when humans display declarative gestures, it is taken to index social goals that are absent from the gestural communication of great apes, but this begs the question of the nature of the evidence for those same goals in the communicative gestures of preverbal humans, who cannot assert those goals; this constitutes a double standard of proof. Thus, in summary, their argument reduces the strong, biconditional position to the weaker, conditional position—because a “truly” declarative act now is defined by the presence of a concomitant motivational state that has no unique behavioral index. In effect, Carpenter and Call (2013) have argued that a communicative act can be ambiguous as to its mental causes, and this constitutes a concession that hypothetical causal mental states are not actually necessary, but merely sufficient causes. Thus, even if ape–human comparisons were methodologically rigorous, there is no unique, nonverbal behavioral index of any alleged causal mental state (Povinelli and Giambrone 2001).

## Mental states as sufficient causes (conditional)

This weaker, and more popular, version of the mental causality model is the idea that certain mental states are sufficient, but not necessary to the display of certain communicative acts. This is the position of Povinelli et al. (2000): “the exact same behaviors can be produced [both with] and without… explicit representation of mental states” (2000, p 533). According to this weaker version, organisms can succeed in experimental tasks like those listed in Table 1 in one of at least two ways: either (a) the organism has a cognitive capacity that causes their own response patterns to the experimental challenges (*p*)—that is, they understand that seeing by others leads to others’ knowing, that others have particular communicative intentions, that others can have false beliefs, or that the organism and another can have a conceptual common ground—or (b) the organism has acquired some kind of rule-based response pattern based on cues associated with the conceptual factors listed in Table 1, albeit without the conceptual understanding—in other words, the organism can acquire correct response patterns through allegedly simpler, non-conceptual learning mechanisms (which, for simplicity in exposition, we will designate ~*p*; see Table 4 for the specific argumentation).

**Table 4:** Conditional mental causality models and their implications for comparative cognition

Assume *p* exists			The reality: *p* is indeterminable		
*p*	*q*	*p* ⇒*q*	*p*	*q*	*p* ⇒*q*
Conditional (*p* is a sufficient cause *q*; *p* implies *q*)					
T	T	T	T?	T	?
T	F	F*	T?	F	?
F	T	T	F?	T	?
F	F	T	F?	F	?

Under the assumption, at left, that *p* is determinable (i.e., that the presence and absence of *p* is determinable, hence a truth value can be legitimately applied to both *p* and *q*), the asterisk denotes the state of the world that would disconfirm the premise *p* ⇒*q*; in this case, when a causal mental state (*p*) does not result in the implied behavior (*q*). Here we argue that, in reality, because *p* is imaginary and cannot be measured, as shown at right, therefore there is no possibility of disconfirming the premise *p* ⇒*q*. Thus, all mental causality models that posit certain mental states (*p*) to be sufficient causes for certain behaviors (*q*) are unfalsifiable. Note that precisely the same reasoning applies to the contrapositive: ~*q* ⇒ ~*p*. In the cases of the inverse (~*p* ⇒ ~*q*) and the converse (*q* ⇒ *p*), the truth tables are slightly different, such that the conditional is falsified when *q* is true (present) and *p* is false (absent), but the general argument that the conditional is unfalsifiable in the case of an imaginary *p* holds

If these mental states are merely sufficient causes of overt behavior, then the relationship between putative mental capabilities and behavior is, by definition, a conditional relationship: if such and such a mental state (*p*) is present in the mind of the subject then they will display such and such a behavior (*q*). In the contemporary literature, there is a near-universal commitment to the idea that behavior does not uniquely implicate a precipitating epistemic mental state; thus, a given response pattern (*q*) could result from the effects of particular hypothetical mental causes (*p*) or from some other psychological process that does not involve these precipitating mental causes (~*p*: e.g., Povinelli and Giambrone 2001; Povinelli et al. 2000; *contra* Tomasello et al. 2007). Thus, when any given response pattern (*q*) can be caused by alleged mental state reasoning (*p*) and also by learning processes in the absence of hypothetical mental state reasoning (~*p*), then the objectively measurable responses of organisms can give no insight into the psychological causes of behavior.

This theme of sufficiency but not necessity suffuses the contemporary literature in comparative cognition: It has become almost a universal practice to report whether or not the organisms under consideration have learned to respond differentially over the course of an experiment. The significance of this is that contemporary researchers almost universally, albeit often implicitly, acknowledge in their scientific practice that a given behavior pattern (*q*) does not uniquely implicate a causal mental state (*p*) because procedures are adopted to clarify whether the organisms’ response patterns were in place before the experiment or were acquired in the course of the experiment. In short, there is a general agreement that any particular response pattern (*q*) can emerge as a consequence of a hypothetical inferential or other deductive hypothetical psychological process (*p*) or by some non-deductive, non-inferential, relatively simple learning process, such as classical or operant conditioning (~*p*). This assumption is, however, not warranted.

Theoretically, it could be the case that an inferential causal process (*p*) is, itself, the product of a learning process (*r*)—sufficient experience with appropriate feedback (response consequences) could lead to a generalized response pattern (*q*). In practice, most contemporary researchers incorrectly take all circumstances in which animals learn through experience (*r*) to discriminate the relevant stimuli as evidence against *p* (see, e.g., Reddy and Morris 2004); this was precisely the argument of Povinelli and Eddy (1996) when their chimpanzee subjects displayed higher performance over time and with experience in their experimental protocols. However, there is no compelling a priori reason to contrast learning through experience (*r*) with hypothetical causal mental states (*p*). If a generalized response pattern (*q*) consistent with, say, an understanding of visual attention (*p*) emerges after sufficient task-relevant experience (*r*), then it would be legitimate to argue that correct responding (*q*) implies an understanding of visual attention (*p*) because this task-relevant experience (*r*, be it an intentionally administered training protocol or simply developmental experience with the appropriate contingency structures) is, itself, a sufficient condition for the hypothetical causal mental state (*p*). Under this framework, when apes do not display correct choice behavior when given nonverbal cues to a baited container (~*q*), it is a legitimate conclusion that they lack a deductive or inferential understanding of visual attention (~*p*) and it is also valid, in this framework, to argue that they additionally lack the appropriate task-relevant experience (~*r*). According to the argument we are advancing, here, given that learning experience can be, in principle, objectively measured, then no account of cognitive performance is complete in the absence of an understanding of individual learning history. In many cases, particularly with long-lived organisms, much is unknown about individual learning histories, but this uncertainty about the degree of task-relevant learning experience must be explicitly acknowledged in all interpretations of socio-cognitive performance. In addition, if performance can be predicted by learning history, then there is no need to appeal to hypothetical, invisible psychological variables in the interpretation of performance. In fact, many contemporary critiques of the many published claims that animals lack the kinds of causal psychological processes listed in Table 1 take this general approach (e.g., Bard and Leavens 2014; Gardner 2008; Leavens 2006; Leavens et al. 2008; Lyn 2010; Lyn et al. 2010; Racine et al. 2008; Russell et al. 2011). Hence, the widespread assumption that hypothetical, causal, inferential or deductive mental states (*p*), on the one hand, and the operant learning through experience (*r*), on the other hand, constitute mutually exclusive kinds of causes of correct choice behavior in discrimination tasks is unwarranted—it could be the case that a generalized deductive process emerges given adequate learning opportunities (e.g., Rumbaugh et al. 1996). More succinctly, the assumption that the existence of task-relevant learning categorically excludes hypothetical acts of reason (if *p* then ~*r* AND if *r* then ~*p*) is, itself, unfalsifiable, due to the objective impossibility of demonstrating the presence or absence of an invisible causal mental state *p*.

These considerations constrain the range of valid conclusions that can be drawn from ape–human performance comparisons, specifically with respect to the use and comprehension of communicative gestures, but also more generally in the domain of social cognition. Significantly, the premise of a conditional relationship between *p* and *q* can only be falsified if we find *p* (a hypothetical mental cause) in the absence of the predicted behavior pattern *q*—as we have already noted, it is just as impossible to empirically measure the presence of a hypothetical causal mental state as it is to empirically measure demonic influences. The reader can easily test the validity of our claim by simple substitution. Examining Table 1, for each of the hypothetical psychological constructs under the column heading, “Putative mental state (*p*),” substitute for that cognitive capability, “the influence of a demonic spiritual agent.” Thus, where Povinelli and Eddy (1996) argued that the conceptual understanding that seeing leads to knowing will lead to high performance in their experimental tasks (where, typically, organisms chose between one of two experimenters), we are going to argue, here, that when organisms are influenced by demonic spiritual agents (*p*), then they will choose the experimenter who can see them (*q*) more than would be expected by random choice behavior. Let us take Povinelli and Eddy’s findings at face value: The human children chose the experimenter who could see them, whereas the apes did not.^5^ It is valid to conclude from this pattern of results that the apes were not under the apparent influence of demonic spiritual agents. It cannot follow that the children were under such an influence—that would be a fallacious conclusion, an attempt to argue from the consequent to the antecedent. Thus, the major premises of these weaker, conditional mental causality models (if *p* then *q*) are unfalsifiable, in principle, as are the stronger biconditional versions (if *p* then *q* AND if *q* then *p*).

To summarize: If we are given, on theoretical and empirical grounds, the following framework: (a) Epistemic states exist prior to choice behavior (e.g., Malle and Hodges 2005); (b) epistemic states are not uniquely specified in choice behavior (e.g., Povinelli and Giambrone 2001); (c) epistemic states cause choice behavior, and (d) it is impossible to directly measure mental states (Bergmann 1962; Vanderwolf 1998), and then mental causality models are unfalsifiable, in principle. An entailment of this structural unfalsifiability is that these mental causality models also cannot implicate the presence of any of the hypothetical mental causes in any species, including humans. It is not rational to conclude the presence of *p* from the presence of *q*—this is a well-known logical fallacy, Affirmation of the Consequent. Hence, if one believes that certain mental states (or demons, or angels, or spirits, or what have you) will cause organisms to point declaratively (e.g., Carpenter and Call 2013), the display of a declarative point cannot be legitimately taken to be evidence for the alleged causal mental state (or any other imaginary cause). Suppose that one’s major premise is that if it rains Sue will get wet. If we find Sue to be wet, it does not follow that it had rained, because there are so many other ways in which Sue could have become wet. Because all of the studies in Table 1 take precisely this logical form, therefore none of the studies in Table 1 provides any evidence that young humans act in accordance with the putative mental state conceptions that the researchers claimed caused their behavior. Thus, the two-group, two-species comparison cannot, by its very design, illuminate the cognitive underpinnings of organisms’ understandings of social behavior (Bard and Leavens 2014).^6^ These studies can only assert that the groups tested performed differently, but are unanimously mute on why that may be (see Racine et al. 2008, 2012).

At best, on purely logical grounds, researchers can deny that non-humans make choices in their environment informed by hypothetical mental state concepts but these studies cannot, in principle, demonstrate that these hypothetical concepts had any role, whatsoever, in the behavior of organisms, usually young humans, who do act in accordance with the theoretical stipulations that particular mental states cause particular response patterns.

## Beyond unfalsifiable psychologies of communication

None of the studies in Table 1 constitutes a scientifically legitimate claim for uniquely human communicative motivations or cognitive processes. This is for two reasons: one methodological and one logical. On methodological grounds, there is no published demonstration of a response difference in any direct ape–human comparison that is not confounded with lurking variables, such as those listed in Table 1. We are not the only researchers to have noted these methodological infelicities (e.g., Boesch 2007; 2012; Bulloch et al. 2008; Gardner 2008; Kellogg and Kellogg 1933; Lyn et al. 2010; Pedersen et al. 2009; Racine et al. 2008, 2012; Russell et al. 2011; Scheel et al. 2017), yet two-group, two-species comparisons persist in the contemporary scientific literature.

On logical grounds, all claims of unique human psychological capacities to represent the mental states of others suffer from either unfalsifiability (no independent measure of the alleged psychological capacities; e.g., Leavens et al. 2004b) or fallacious reasoning (taking a discrimination, *q*, as evidence for a particular mental cause, *p*, of that discrimination). Thus, there is no logical pathway from overt, publicly available behavior to any claim of the causal influence of any particular hypothetical mental state. Note that there are cognitively relevant scientific hypotheses that can be empirically tested. For one example, as noted above, scientists have tested whether intensive exposure to a linguistic environment would produce an ape that speaks, and the answer seems to be that mere exposure to speech (*p*) is not sufficient to produce speech (~*q*) in apes (e.g., Kellogg 1968)—the major premise that speech exposure causes speech production (if *p* then *q*) has been falsified—this is a true species difference between apes and humans. Similarly, it is possible to ask whether intensive exposure to human cultural environments (*p*) can cause the use of manual pointing gestures (*q*) in great apes: here, the answer seems to be in the affirmative—all apes raised in unusually close contact with humans (i.e., enculturated apes), without a single exception, demonstrate pointing behavior—there is, to date, no example of an ape who has been enculturated (*p*) but who does not point (~*q*).^7^ Unlike hypothetical, invisible mental causes, such antecedents as these examples—speech-intensive early rearing environment or intensive exposure to human conventions of nonverbal signaling—are empirically measurable, and hence scientific hypotheses about relationships between antecedents and consequents are, in principle, falsifiable. Therefore, our general conclusion is that mental process models that incorporate imaginary antecedents are fatally unanchored in objective reality, therefore of little or no utility in scientific hypothesis testing (Leavens et al. 2008, *fn* 2).

In the absence of appropriate experimental designs and adequate deductive methods, it is reasonable to ask whether comparative or developmental psychology has anything useful to contribute to our understanding of what is frequently termed the “cognitive foundations” of communication development. We think, first, that the ambition is over-blown for at least two reasons. First, in our judgement, claims of illuminating the “cognitive underpinnings” of communicative behavior in preverbal humans, great apes, and other animals are inflated to the very degree that aspects of “cognitive underpinnings” are hypothetical—if both cherished theoretical constructs such as discernment of false belief, a motivation to share feelings or epistemic states, or an understanding of visual attention, on the one hand, and a history of task-relevant experience with an appropriate reward-contingency structure, on the other hand, can result in an organism that can make generalized discriminations of the kinds employed in the studies listed in Table 1, then the state of reality is that these two classes of cause (mental state versus operant history) do not make contrasting predictions about behavior. Moreover, as we argued above, mental state causes and learning of social contingencies through experience are not necessarily mutually exclusive antecedents. In addition, as we have illustrated (Table 1), no researcher has ever isolated evolutionary history as a factor in ape–human differences in published assays of comparative social cognition involving direct ape–human comparisons, particularly the social cognition of communication; the reason for this systematic design failure is attributable to the difficulty of properly matching groups sampled from different species. Our argument, here, is that this failure to match on so many crucial life history and procedural variables should not continue to be ignored in the contemporary literature. Finally, according to our analysis, where responses to cognitive challenge are viewed as the effects of hypothetical, invisible causes, as in a substantial fraction of work in this area, it is both logically and empirically impossible to demonstrate the influence of these same hypothetical causes. We think that there are at least four productive approaches to comparative social cognition that avoid these systemic problems: cross-fostering of apes by humans; radical operationalization; training; and sampling across the full ecological range of a species.

## Cross-fostering

Cross-fostering of apes by humans has a long history (Gardner and Gardner 1969; Hayes and Hayes 1954; Hillix and Rumbaugh 2004; Hoyt 1941; Kearton 1925; Kellogg and Kellogg 1933; Ladygina-Kohts 1935; Matsuzawa 1985; Miles 1990; Patterson and Cohn 1990; Premack and Premack 1972; Rumbaugh 1977; Savage-Rumbaugh 1986; Savage-Rumbaugh et al. 1978; Temerlin 1976; Witmer 1909). As noted by Kellogg (1968):

Although often misunderstood, the scientific rationale for rearing an anthropoid ape in a human household is to find out just how far the ape can go in absorbing the civilizing influences of the environment. To what degree is it capable of responding like a child and to what degree will genetic factors limit its development? (p 426).

We note that, from a purely methodological point of view, these cross-fostering studies ameliorate, to varying extents, the incommensurate individual learning histories that apes and humans typically bring to experimental challenge. Astonishing insights into the capabilities of our nearest living relatives to comprehend spoken or signed language (e.g., Gardner and Gardner 1969; Rumbaugh 1977; Savage-Rumbaugh et al. 1993), and to produce symbolic communication have been demonstrated by the classic cross-fostering studies (e.g., Gardner and Gardner 1969; Hayes and Hayes 1954; Kellogg and Kellogg 1933; Savage-Rumbaugh et al. 1978; Patterson and Cohn 1990). These studies have repeatedly shown, for example, that apes do not produce even modest repertoires of spoken language, even when given exposure to broadly the same linguistic input as human children (Kellogg 1968). Yet, these cross-fostered apes do produce species-atypical communicative competencies. For example, Chantek, a sign-language-trained orangutan (*Pongo pygmaeus*) has displayed highly competent comprehension of pointing (Call and Tomasello 1994; Tomasello et al. 1997), belying the frequent false claim that great apes have difficulty comprehending these deictic signals—apparently when sufficient task-relevant pre-experimental experience is given to apes (i.e., when apes are matched with human children on this critical variable), then they act more like human children do (e.g., Leavens 2014; Leavens and Bard 2011; Leavens et al. 2010; Lyn et al. 2010; Russell et al. 2011). Cross-fostering of apes by humans has significant ethical implications: some apes who are both cross-fostered and isolated from their conspecifics have experienced particularly grim outcomes, especially when inadequate provision has been made for their long-term psychological well-being (Fouts and Mills 1997). Many contemporary researchers now hold the view that cross-fostering of apes by humans is categorically unethical (Fouts and Mills 1997), although infant apes in zoos are routinely cross-fostered by humans on a temporary basis when their survival is at risk. Consideration of these factors can only highlight the importance of the dwindling populations of cross-fostered apes for understanding environmental influences on cognitive development (e.g., Lyn et al. 2010).

## Radical operationalization

We (e.g., Leavens et al. 2004a, 2005a, 2008) and others (e.g., Bourjade et al. 2015; Townsend et al. 2017) have advocated a radical operationalization of mental state terminology. We believe that, in the absence of any empirically grounded pathway toward clarification of mental causes, the theoretical assumptions of the causal mental state model are scientifically untestable due either to (a) inappropriate ontology (a metaphysical concern) or (b) to the technically immature state of experimental psychology (an epistemological concern; for criticisms of ontology, see, among many others, Barrett 2014; Di Paolo and De Jaegher 2012; Leudar and Costall 2004; and Varela et al. 1991), and for technical limitations; see, e.g., Bergmann 1962, Clark 2001). The central difference between radical operationalization and the mental causality model of psychological processing is that whereas mental causality models view behavior as effects of mental causes, radical operationalization views mental states as being sufficiently defined by behavior and context. No organism, A, can base an evidenced judgement about the motivation of another organism, B, in the absence of (a) B’s physical behavior, (b) the physical antecedents of B’s behavior, or (c) the physical consequences of B’s behavior; in the absence of supernatural causes, all mental states must be expressed in physical terms (Leavens et al. 2008). Therefore, it is arbitrary to exclude objectively measurable physical aspects of an organism’s comportment in the world from the category of mental states—mental states are embodied as much in our muscles, our skeletons, and our artifacts as in our brains (e.g., Barrett 2014; Brinck 2014; Johnson 2001). Thus, with respect to intentional communication, human babies are said to communicate intentionally when they act *as if* they have goals in advance of signaling, when they tactically accommodate their signals to the attentional availability of an interlocutor, and when they act to manipulate the visual focus of an interlocuter—all of these patterns are empirically discoverable, in any species (Leavens et al. 2005a; Townsend et al. 2017). This approach will not reveal hypothetical psychological causal factors, but as we’ve argued, here, this incapacity is inherent in all contemporary approaches to comparative and developmental social cognition. Moreover, there are exciting new theoretical approaches to cognition that reject mental causality (e.g., Barrett 2015; Barrett and Henzi 2005; Bateson 1972; Baum 2002; Chemero 2011; Johnson 2001; Rachlin 1992; Shanker and King 2002; Varela et al. 1991) in a variety of different ways that need not concern us here; the significant fact of these alternative theoretical approaches, for present purposes, is that they are not subject to the same scientific problems outlined in the preceding pages in relation to the mental causality model (Leudar and Costall 2004; Sehon 2000).

## Training

We (Bard and Leavens 2014; Hopkins et al. 2013; Leavens 2006, 2014; Leavens et al. 2008, 2015; the present analysis) and others (e.g., Boesch 2007, 2010, 2012; Gardner 2008; Kellogg 1968; Rumbaugh et al. 1996) have noted that, in general, many contemporary, cross-species tests of social cognition involve the testing of apes on their understanding of culturally situated human conventions of gestural signaling (e.g., pointing or gazing to the location of hidden entities). Because most of these apes lack exposure to these communicative conventions, particularly in relation to the Western human children with whom they are being compared, therefore, it would be scientifically productive to train non-humans for long periods with experiences designed to foster the discriminations that are used to test subjects’ social cognition. Consider, for example, that it takes human children approximately 9 months from birth to follow a pointing gesture to a nearby location and almost twice that long to follow a pointing gesture to a location behind themselves (Butterworth 2003). If we find that an organism fails to follow a pointing gesture with less than 18 months of comparable exposure (as did Povinelli et al. 1997 and Tomasello et al. 1997, among others), then it cannot follow from this that the species lacks the cognitive capacity for this comprehension because the organism has been handicapped by lack of task-relevant pre-experimental experience, relative to human children. Yet many researchers continue to claim that apes are cognitively deficient, relative to humans, when representatives of apes are exposed to these signals for a few minutes in an afternoon or two (Leavens 2006), or exposed at much reduced absolute or relative frequencies (Thomas et al. 2008).

We believe that task-relevant and extended training protocols for passing these kinds of socio-cognitive assays with apes have great promise in illuminating the factors in the environments of human babies that foster communicative development in our own species. We note that many contemporary researchers reject this idea because they argue that humans display these competencies “spontaneously” (e.g., Bohn et al. 2015, 2016; Carpenter and Call 2013; Povinelli et al. 2003; Warneken et al. 2007), but as we see it, usually when a behavioral scientist claims that a capability is displayed “spontaneously,” this is tantamount to a confession that the ontogenetic pathway to that capability is not known—it cannot be taken as evidence that the behavior of interest has no developmental history, nor can “spontaneous” exhibition of a behavior constitute evidence that this behavior has no learned basis.^8^ Again, if apes are isolated from a suite of specific ecological factors in early development to which human children are intensively exposed, then it is naïve to assume that these factors had no influence on the later “spontaneous” display of particular competencies by the human children. Only in the case that organisms do not display a competency after very extensive training protocols designed to facilitate those competencies can the hypothesis of species incapacity be legitimately entertained (Leavens et al. 2015).

## Sampling

It is now well-understood that psychology has been overly reliant on Western, educated, industrialized, rich, and democratic (WEIRD: Henrich et al. 2010) samples to represent the entire human species, and we have observed that similar biases exist in the sampling of great apes and other non-human comparison groups in comparative psychology (Bard and Leavens 2014; Hopkins et al. 2013; Leavens et al. 2005b, 2010; also see Boesch 2007, 2010, 2012). There are systematic differences in performance on cognitive assays between groups of representatives within species, and these can be manifested very early in development (Bard et al. 2014a, b). Thus, a fruitful avenue of research for comparative psychologists is to compare, within-species, groups that have experienced systematically different early rearing experiences (e.g., Bard et al. 2014a, b; Call and Tomasello 1994; Lyn et al. 2010; Pitman and Shumaker 2009; Rumbaugh et al. 1996; Russell et al. 2011). A post hoc approach, sampling the range of phenotypic variation within a species, can identify environmental plasticity, paving the way for further exploration of specific environmental factors (e.g., Leavens et al. 2010). Because almost everything we know about human cognitive development also rests on post hoc sampling of different groups, for good ethical reasons, such sampling does not constitute a methodological weakness, relative to our sampling of humans. A second challenge of this approach is the sheer difficulty of acquiring appreciable samples of subjects for study across the range of rearing environments, but we note that even very small-sample studies can produce extraordinarily large effects, when carefully conducted—for example, Call and Tomasello (1994) reported large performance differences in the use and comprehension of pointing gestures between two orangutans, tested in similar protocols in the same testing environment, that clearly demonstrated a lack of parity in their responses to the cognitive challenges administered in that study.

## Conclusions

On both methodological and logical grounds, the mental causality model of psychological processes has failed to produce any unambiguous ape–human differences in social cognition. Despite numerous claims to the contrary, no current scientific methodology has isolated evolutionary history as the causal factor in alleged ape–human differences in social cognition. Moreover, every such claim of a “species difference” has been refuted by superior methodological approaches, involving within-species explorations of specific competencies (see notes to Table 1). Thus, where differences have been reported between ape and human groups, the relevant factors accounting for these differences (environmental, genetic) remain unknown. Thus, to claim a “species difference” in social cognition between apes and humans, at our present state of knowledge, is to promulgate the same kinds of prejudices that hereditarians evinced in the early history of biometric approaches to the study of intelligence—all group differences were taken to be evidence for innate, primary differences in abilities between different groups of humans, and environmental influences on mental development were routinely ignored (Gould 1981). Tables 1 and 2 document the same sort of wishful thinking (systematic bias) in the face of the numerous confounds listed there.

On logical grounds, the existence of hypothetical, causal mental states cannot be confirmed, with present technology. Hence, there is no evidence that the communicative signaling of humans, great apes or other animals, is predicated on substantially different cognitive bases. Current psychological process models that emphasize the allegedly causal nature of imaginary, invisible psychological processes like those listed in Table 1 are unfalsifiable, for several reasons, but primarily because no putative causal mental state, to date, is uniquely specified by any particular behavior pattern. By rudimentary logical principles, therefore the existence or effect of these imaginary, alleged psychological causal mental factors cannot be demonstrated by appeal to particular behavioral response patterns.

The field of comparative psychology could benefit from greater attention to the ecologically situated competencies of all research subjects/participants—especially cross-fostered animals, a greater commitment to operationalization of mental state concepts, intensive training studies, and sampling of subjects across a wider range of rearing histories. We think that it is especially important not to reject, out of hand, relevant evidence from populations that might differ in important ecological circumstances from one’s own study population. Thus, if we study institutionalized representatives of a species of ape and we find that they systematically differ in their communicative behavior from reports of their conspecifics who have been cross-fostered by humans or conspecifics living in wild habitats, we believe that it is more scientifically informative and fruitful to take these differences as signposts to ecological factors that might influence communication development, than it is to reject the evidence outright on such grounds as, for example, that the animals are raised in unnatural circumstances, or that those studying wild populations cannot adequately control the circumstances of their observations (Bard and Leavens 2014; Leavens et al. 2010).

The central message of this analysis for researchers interested in the origins of language is that too strong a focus on specific communicative behaviors (e.g., pointing with the index finger, declarative-expressive communication, vocal signals with apparent semantic content, and so on) without proportionate attention to (a) the contextual influences on subjects’ responses (e.g., Hopkins et al. 2007; Leavens et al. 2005a, 2010; Schel et al. 2013), (b) the developmental course of these communicative signals (e.g., Bard and Leavens 2014; Bard et al. 2014a, b), (c) the concomitant bodily correlates of the signals (e.g., Leavens and Hopkins 2005; Slocombe et al. 2011), and (d) the range of variation in communicative repertoires within species (e.g., Hobaiter and Byrne 2011; Leavens et al. 2010; Roberts et al. 2014; Wich et al. 2008, 2012) will too easily—and inaccurately—load communicative and theoretical cognitive competencies on the human lineage, after our split from our nearest living relatives. Like humans, other animals develop their communicative repertoires in specific ecological contexts, and their signaling adapts, ontogenetically, to these specific environmental circumstances. Sensitive attention to how animals deploy their signals in relation to these local ecological challenges, with consideration of their specific individual learning experiences (i.e., systematic patterns of response consequences), will illuminate the true range of communicative competencies in any given species. To give an example from our own research, we find that when the environment provides a function for communicating with deictic gestures (referential signaling), then chimpanzees will display referential gestures (e.g., Leavens et al. 1996, 2005a), despite the extreme rareness of pointing in chimpanzees’ wild habitats (Hobaiter et al. 2014), and similar results have been reported for a range of non-human primates including orangutans (Cartmill and Byrne 2007; Miles 1990; Pelé et al. 2009) and gorillas (*Gorilla gorilla*, Tanner et al. 2006). Thus, the capacity for gestural reference results from the dynamic dialectic of interaction between organisms and their specific, lived ecological configurations, and we might therefore reasonably speculate that the last common ancestor of apes and humans had a latent capacity for referential signaling that is particularly adaptive, in ontogenetic terms, in WEIRD human rearing environments. This environmentally based behavioral variation in signaling behavior by groups of animals sampled from the same gene pool authorizes the search for those particular ecological factors that support referential communication during development (Leavens et al. 2005b). This approach is inherently more fruitful than to compare differently aged representatives of humans and great apes with virtually no experimental control over task-relevant pre-observational experience, incommensurate sampling protocols, and often radically different test procedures—an investigative approach that we are condemning in this paper: any differences that emerge in response profiles between groups compared in this makeshift manner will never constitute evidence for some kind of “key” cognitive adaptation for communication in the human lineage (Bard and Leavens 2014). Rather, the only firm conclusion that can be made is that apes not raised in western, postindustrial households do not act very much like human children who were raised in those specific ecological circumstances, a result that should surprise no one. The two-group, two-species ape–human comparison is scientifically untenable; we present four methodological remedies to the mismeasure of ape social cognition.

## Footnotes

1 Lest some readers doubt the veracity of this claim: we attempted to teach children to point in the same manner as the chimpanzees, but most children were extremely reluctant or embarrassed to come forward and point at the experimenters. In addition, *the points that they did produce were often so subdued that it was difficult to determine exactly to whom they were referring*. In contrast, the children rapidly adapted to the procedure of extending their arm and placing it on the handprint in front of the target experimenter in order to obtain their rewards (stickers). (Povinelli and Eddy 1996, fn 6, p 109, emphasis added).

2 For simplicity in argumentation, we include as causes alternative formulations in which certain mental states may merely facilitate or make more probable correct responses patterns in a cognitive challenge. As an anonymous reviewer pointed out, other possibilities exist: For example, it could be that correct performance in a discrimination task is necessary, but not sufficient condition for the presence of causal mental states (*q* ⇒ *p*), which is the logical converse of stating that *p* is sufficient for *q*. In this case, the converse of *p* ⇒ *q*, the mental causality model imposes an asymmetry that does not exist in purely inferential logic—scientific explanations seek to account for a behavior in terms of the actions of hypothetical, temporally prior causes. This temporal asymmetry does not necessarily exist in purely formal contexts—indeed, the converse relationship that exists between sufficiency and necessity in logic is only legitimate when it is not the case that *p* and *q* exist in a fixed, non-overlapping temporal order, as they do in mental causality models. Thus, while it is a correct rule of inference that if *p* is sufficient for *q* to be the case then *q* is necessary for *p*, this relation between sufficiency and necessity breaks down when the context is explanatory, with a fixed, asymmetrical relation between *p* and *q*, as in the present context. Hence, the converse (*q* ⇒ *p*) will not be further considered here. In addition, it might be that mental states are neither necessary nor sufficient causes for high performance in a discrimination task (i.e., that *p* and *q* have no causal relationship), because our concern, here, is to highlight the inability to empirically test the truth of models of alleged mental causality, therefore this lies beyond the scope of this paper.

3 Thanks to an anonymous reviewer for bringing Baum’s and Rachlin’s work to our attention.

4 There are standard methods for the determination of demonic possession, both historically (Kors and Peters 2001) and in the present day (e.g., MinisteringDeliverance.com). For example, “[a]rthritis, cancer, HIV, and a host of other illnesses are almost always caused by evil spirits. If it cannot be easily cured, then it's almost always a spirit that is causing it” (http://www.ministeringdeliverance.com/demonic_signs.php); this claim constitutes a claim that demonic possession is a sufficient cause of these diseases. For an example of a necessary and sufficient cause:, a “man may very surely be known for a demoniac if he is disturbed when the exorcisms are read” (Guazzo, 1608/1988, p. 168); this claim is of precisely the same form as the claim that we know that humans possess unique psychological motivations for communication when they display declarative-expressive or declarative-informative communication.

5 This is a simplification: Actually the apes performed very well in most conditions, either from the outset or with a modicum of experience (see Povinelli and Eddy 1996).

6 There are other possible outcomes of any given ape–human comparison: It could be the case that there are no systematic group performance differences; in this case, under the mental causality model, one can only conclude that there is no evidence of group differences in hypothetical causal mental states—positive conclusions to the effect that the subjects are motivated by the same hypothetical causal mental states are unwarranted.

7 One enculturated ape, Viki, was reported to point to nearby objects, but not distally (Hayes and Hayes 1954).

8 Thanks to Roger K. Thomas for this observation (Leavens 2015).
